# Asymmetric recurrent laryngeal nerve conduction velocities and dorsal cricoarytenoid muscle electromyographic characteristics in clinically normal horses

**DOI:** 10.1038/s41598-019-39189-z

**Published:** 2019-02-25

**Authors:** Marta Cercone, Caitlin M. Hokanson, Emil Olsen, Norm G. Ducharme, Lisa M. Mitchell, Richard J. Piercy, Jon Cheetham

**Affiliations:** 1 Department of Clinical Sciences, College of Veterinary Medicine, Cornell University, Ithaca, NY USA; 2 Comparative Neuromuscular Diseases Laboratory, Department of Clinical Sciences and Services, Royal Veterinary College, London, NW1 0TU UK

## Abstract

The dorsal cricoarytenoid (DCA) muscles, are a fundamental component of the athletic horse’s respiratory system: as the sole abductors of the airways, they maintain the size of the rima glottis which is essential for enabling maximal air intake during intense exercise. Dysfunction of the DCA muscle leads to arytenoid collapse during exercise, resulting in poor performance. An electrodiagnostic study including electromyography of the dorsal cricoarytenoid muscles and conduction velocity testing of the innervating recurrent laryngeal nerves (RLn) was conducted in horses with normal laryngeal function. We detected reduced nerve conduction velocity of the left RLn, compared to the right, and *pathologic spontaneous activity* (PSA) of myoelectrical activity within the left DCA muscle in half of this horse population and the horses with the slowest nerve conduction velocities. The findings in this group of horses are consistent with left sided demyelination and axonal loss, consistent with Recurrent Laryngeal Neuropathy (RLN), a highly prevalent degenerative disorder of the RLn in horses that predominantly affects the left side. The detection of electromyographic changes compatible with RLN in clinically unaffected horses is consistent with previous studies that identified “subclinical” subjects, presenting normal laryngeal function despite neuropathologic changes within nerve and muscle confirmed histologically.

## Introduction

Horses are highly developed athletes, able to reach a maximum speed approaching 88 Km/h with a maximal oxygen consumption of 200 ml/kg/min^1^. Despite marked cardiovascular and musculoskeletal adaptations, the respiratory system represents a common limiting factor for oxygen delivery and athletic performance^1–3^. Dilation of the rima glottis during exercise is a key, often rate limiting component of the upper airway patency and is maintained by contraction of the sole arytenoid abductor, the dorsal cricoarytenoid (DCA) muscle^1,4^.

The most common cause of dysfunction of the equine DCA muscle, and reduced athletic performance, is Recurrent Laryngeal Neuropathy (RLN). This disease is characterized by loss of large, alpha myelinated nerve fibers in (predominantly) the left distal recurrent laryngeal nerve (RLn)^5–9^. The majority of the histological changes detected (Büngner bands, regenerating clusters, paranodal evaginations, and spheroids) are associated with primary axonal dysfunction and pathological features usually associated with primary myelinopathies (onion bulbs and demyelination), but that also occur in primary axonopathies (for the most recent review, see Draper and Piercy, 2018)^10^.

The aim of this study was to conduct a comprehensive electrodiagnostic study of the DCA muscles and recurrent laryngeal nerves to gather normative data in horses with normal laryngeal function at rest and during exercise. We performed needle electromyographic evaluation of the left and right DCA muscles during spontaneous breathing and following electrical stimulation of the RLn, with measurement of nerve conduction velocities bilaterally.

## Methods

This study was performed in accordance with the PHS Policy on Humane Care and Use of Laboratory Animals, federal and state regulations, and was approved by the Cornell University Institutional Animal Care and Use Committee (IACUC).

### Animals

Horses were included in the study if healthy on physical examination and showing normal laryngeal function on endoscopic examination both at rest (Havemeyer scale grade I) and during exercise (grade A)^11^. The Havemeyer grading system evaluates laryngeal function based on symmetry of movement of the arytenoid cartilages, describing 4 grades with sub-grades: grade I indicates synchronous and symmetrical movements with complete arytenoid abduction, while grade IV is assigned to horses with complete immobility of the arytenoid and vocal cord. During exercise the evaluation includes 3 grades: grade A is assigned to horses with full arytenoid abduction maintained throughout the exercise, whilst grades B and C are assigned to horses with partial abduction and collapse of the arytenoid. Horses were excluded if they had a history of respiratory noise, laryngeal function grade II or higher at rest, and/or grade B or C during exercise.

In each horse, electromyographic (EMG) activity of the dorsal cricoarytenoid muscle (DCA) was recorded at rest and the RL nerves were localized *in vivo* using an electrophysiologic method designed based on the technique previously described by Steiss *et al*.^12^. Nerve conduction velocity (NCV) of the RLn was calculated using the data collected.

### Electromyography

EMG examinations were carried out with a Sierra^®^ Wave^®^ portable system (Cadwell Laboratories Inc, Kennewick, WA, USA). The EMG signal was filtered with a notch filter at 60 Hz and a band-pass filter allowed frequencies between 30 Hz and 10KHz. The sweep speed was set to 10 ms per division and the amplifier gain to 100 µV per division.

All animals were restrained in a stock and sedated with an alpha-2 agonist (detomidine 0.01 mg/kg), injected intravenously. Detomidine and other alpha-2 agonists do not affect nerve conduction velocity and motor-evoked potentials in humans, and have no effect on the thoraco-laryngeal reflex (which includes the RLn) in horses^13,14^. The laryngeal area was clipped and aseptically prepared on both sides. A monopolar sub-dermal needle electrode was placed as ground electrode in the triangle formed by the caudal aspect of the mandible and the jugular and linguofacial veins, while a recording 23 G bipolar concentric EMG needle (75 mm × 0.65 mm, recording area 0.07mm^2^) (Ambu^®^ Neuroline Needle Electrodes) was inserted percutaneously in the DCA muscle. The right DCA muscle was tested before the left. The muscle was localized by palpation of the laryngeal area after identification of the arytenoid muscular process. The concentric needle was inserted in a dorsolateral-ventromedial direction, at an angle of approximately 60° from the horizontal plane and withdrawn slightly, when the lamina of the cricoid cartilage was reached. The correct position of the needle was confirmed by detection of electromyographic activity during the inspiratory phase^15–17^. The needle position was adjusted to subjectively maximize the signal amplitude, but no attempts were made to record maximally high amplitudes. The needle was re-positioned within the muscle up to three times. EMG activity of the DCA muscle was recorded at rest to include at least 10 respiratory cycles. The procedure was repeated on the left side. Data were collected and stored for later processing.

### Motor Unit Potential (MUP) analysis

An initial qualitative evaluation of the EMG traces was performed blindly by two of the authors (MC, EO) to identify the eventual presence of *pathologic spontaneous activity* (PSA). Electromyographic activity considered abnormal includes spontaneous firing of single muscle fibers (fibrillation potentials or positive sharp waves, myotonic discharges) or a group of muscle fibers (complex repetitive discharges)^18^. The abnormal firing pattern were characterized as PSA if lasting more than 10 seconds or detected in 2 or more traces. Motor Unit Potentials (MUPs) were automatically determined by the EMG software according to an automated template matching method that evaluates similar spikes and sorts them into groups. When a spike has more than 10 matches, it is considered to represent discharges from a single motor unit. The spikes in the group are averaged and the resulting averaged trace was manually corrected by on-screen visual assessment if necessary^19,20^. Each averaged MUP trace was used for automatic quantitative measurements including amplitude (µV), duration (ms), number of phases and turns, firing frequency (Hz) and Size Index. The total percentage of polyphasic MUPs (>4 phases) was also calculated.

### Nerve Conduction study

**Figure 1:**
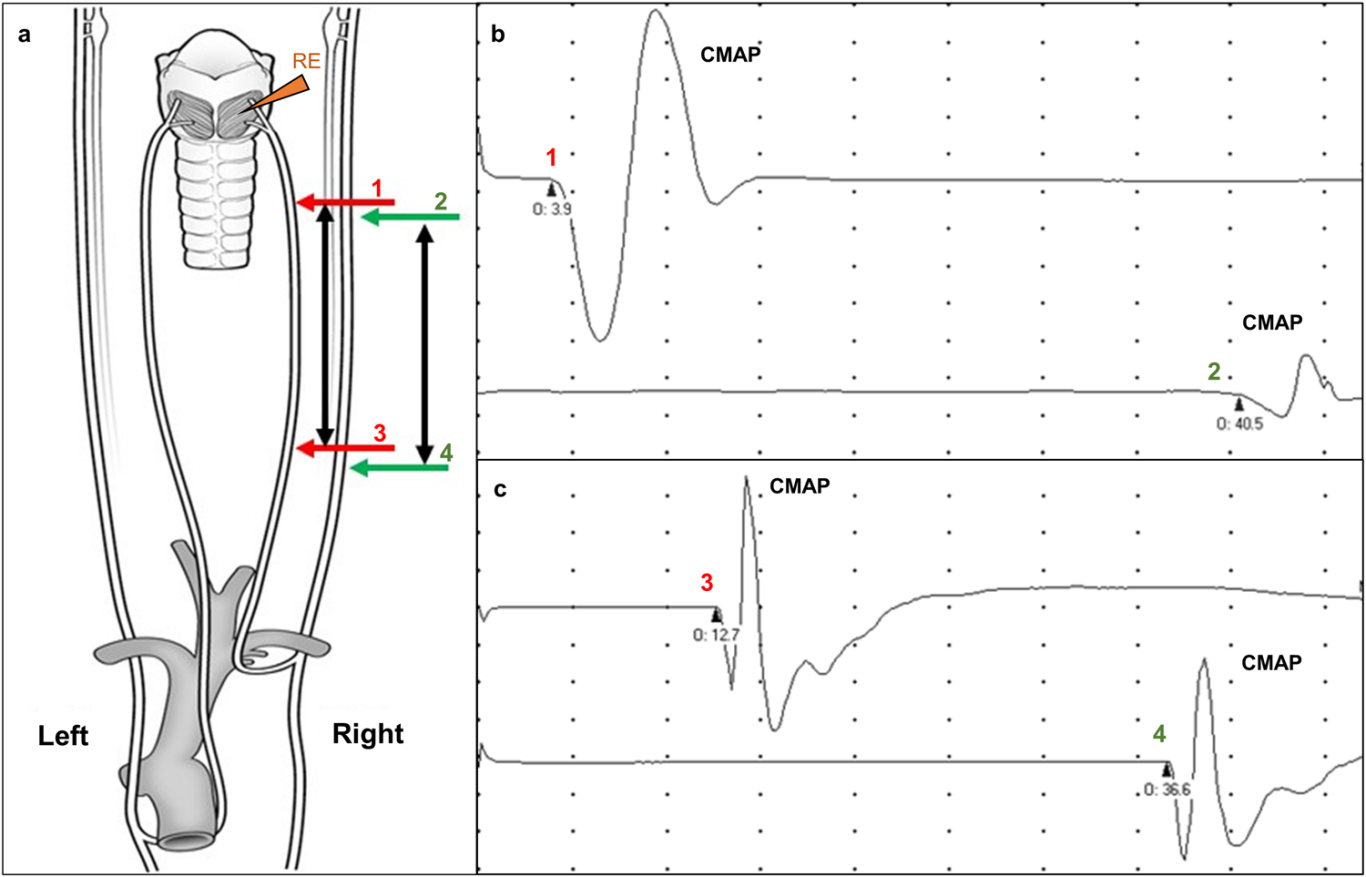
Schematic of the Nerve Conduction study. (**a**) Approach to determine nerve conduction velocity (NCV) in the proximal and distal portions of the equine recurrent laryngeal nerve (RLn). (**b**,**c**) Representative compound motor action potentials (CMAP) detected through the recording electrode (RE) in the dorsal cricoarytenoid (DCA) muscle following supramaximal stimulation of the RLn distally (red arrows) and proximally (green arrows). Differences in onset time (O) used to calculate NCV.

After recording the DCA muscle electrical activity at rest, RLn transcutaneous stimulation was performed, and evoked potentials recorded for subsequent analysis. The RLn stimulation was achieved in two locations along the neck, on each side. The ascending (distal) portion of the RLn is located externally and ventrally to the carotid sheath^21^ and was reached with a monopolar stimulating needle placed dorsal to the jugular vein and perpendicular to the skin in the caudal neck. Supramaximal stimulation at 10 mA and 100 µsec was then applied to produce stimulation of all axons within the RLn and the corresponding compound motor action potential (CMAP) recorded from the DCA muscle. The position of the needle tip was then slightly adjusted to produce supramaximal stimulation of the vagal (proximal) portion of the RLn that bilaterally pass within the carotid sheaths dorsolateral to the common carotid arteries (Fig. 1)^21^. The needle was then repositioned (similarly) to produce stimulation of the ascending and vagal portions of the RLn on the cranial portion of the neck, about 10 cm caudally to the larynx. A minimum of three CMAPs were recorded from each stimulation site. The distance between the EMG recording needle inserted in DCA and the cranial and caudal neck stimulation sites was measured with a measuring tape. The procedure was then repeated on the left side of the neck.

For each CMAP, the latency was determined as the time from the application of the stimulus to the onset of the first waveform deflection from the baseline, representing the conduction of the most rapidly conducting fibers within the nerve segment being tested^22^. The peak-to-peak amplitude was measured on the evoked CMAP on the left and right side. NCV was calculated from the mean difference in CMAP onset time between cranial and caudal neck stimulation of the ascending distal or vagal proximal portions of the RLn and the distance between the two stimulation sites (NCV = distance/difference in CMAP onset time).

### Statistical analysis

Descriptive data are expressed as means with standard deviation (SD). Normal distribution of motor unit potential and nerve conduction study analysis data was assessed using the Kolmogorov-Smirnov test and quantile-quantile (Q-Q) plot. Paired *t*-test or Wilcoxon rank-sum test were used as appropriate for side to side comparison and differences between the ipsilateral distal and proximal RLn portions from each subject. Independent t-test was used for assessing difference between sexes. Pearson’s correlation coefficient was used to determine the correlation between the age and the measured data (NCV, CMAP amplitude, MUP analysis). JMP Pro 12 (SAS Institute Inc.) software was used for the analyses. P values < 0.05 were considered statistically significant.

## Results

Twenty mature horses (mean age 6.5 years ± 3.4, age range 1–16 years; mean height at the withers 157.7 cm ± 5.5, height range 152.4–163.6 cm) of different breed (17 Thoroughbreds, 2 Thoroughbred-Crosses, 1 Warmblood) and sex (8 females, 9 neutered males, 3 males) were included in the study.

### Electromyography

**Figure 2:**
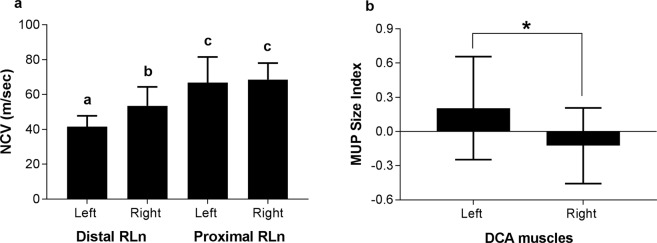
Nerve Conduction Velocity and Motor Unit Potential analysis results. (**a**) Nerve conduction velocity (NCV) is decreased in the longer left recurrent laryngeal nerve (RLn) and in the distal compared with proximal segments of ipsilateral RLn. Different letters indicate statistically significant differences between groups (p < 0.001, n = 20). (**b**) Motor Unit Potential (MUP) Size Index is significantly higher in the left dorsal cricoarytenoid (DCA) muscle (*p = 0.031, n = 20).

Size Index of motor unit potentials (MUPs) was significantly increased in the left dorsal cricoarytenoid muscle compared with the right (p = 0.031, two-tailed paired t-test, Fig. 2) suggesting larger motor unit sizes on the left. Size Index was chosen as representative of MUPs size because, unlike other EMG variables, it is less affected by needle position^23^. Similarly, the duration of MUPs was increased in the left DCA muscle (p = 0.028, two-tailed paired *t*-test) (Table 1)^24^.

**Table 1:** Motor Unit potentials analysis of the dorsal cricoarytenoid muscles.

	Left DCA	Right DCA
Amplitude (uV)	289.64 ± 124.78	230.18 ± 87.81
Duration (ms)	**8**.**85** ± **1**.**48**	**7**.**55** ± **1**.43*
Phases	2.97 ± 0.36	2.95 ± 0.46
Turns	2.28 ± 0.47	2.12 ± 0.5
Frequency (Hz)	5.88 ± 2.12	5.86 ± 2.01
Size Index	**0**.**20** ± **0**.**45**	**−0**.**12** ± **0**.33*

Multi-Motor Unit Potentials analysis of the left and right dorsal cricoarytenoid muscles (DCA). Data are mean ± standard deviation. (*p < 0.05).

Nine horses (45%) demonstrated *pathologic spontaneous activity* in the left DCA muscle, typical of ongoing denervation, and mostly consisting of fibrillation potentials and/or positive sharp waves. In 1 subject, PSA was present in both the left and right DCA. PSA was never observed exclusively in the right DCA muscle. Polyphasic MUPs, (>4 phases), were recorded in 11/20 horses (55%) on the left side (p = 0.0013, two-tailed Fisher’s Exact Test) with 3–17% of MUPs having more than four phases. Animals with polyphasic MUPs were significantly more likely to have *pathologic spontaneous activity* (p < 0.001, two-tailed Fisher’s Exact Test).

The electromyographic data were afterwards grouped based on the presence or absence of PSA, but no significant difference was detected for any morphologic aspect of the MUPs between horses with or without PSA (p > 0.05, independent *t*-test).

### Nerve Conduction Velocity

As anticipated, latency was increased with proximal RLn stimulation compared with distal RLn stimulation, providing clear distinction of a vagal waveform from the distal RLn-evoked compound motor action potential (CMAP) (p < 0.001, two-tailed paired *t*-test, Fig. 1, Table 2). NCV was bilaterally decreased in the distal portion of the RLn compared to the proximal portion running within the vagus (p < 0.05, two-tailed paired *t*-test, Fig. 2, Table 2). We observed a marked reduction in NCV in the distal portion of the left RLn compared to the right (p < 0.0001, two-tailed paired *t*-test, Fig. 2, Table 2) at speeds below the nerve conduction velocity considered normal for mammalian nerve^25–27^. CMAP amplitude was reduced following proximal versus distal stimulation (p < 0.01, 2-sample test, Wilcoxon Signed Rank test), an expected consequence of temporal dispersion of the potentials over an increased conduction distance^22,28–31^. Marked reduction of the CMAP amplitude generated by the distal left RLn stimulation was also detected (p < 0.05, 2-sample test, Wilcoxon Signed Rank test, Table 3)^31,32^.

**Table 2:** Nerve conduction velocity and latency of the recurrent laryngeal nerves.

			Left	Right
NCV (m/sec)		Proximal	66.77 ± 14.74	63.95 ± 14.89
		Distal	**41**.**44** ± **6**.**3**	**53**.**47** ± **10**.9***
Latency (ms)	Cranial neck	Proximal (1)	**33**.**12** ± **3**.**05**	**22**.**9** ± **2**.98***
		Distal (2)	**5**.**12** ± **0**.**89**	**4**.**13** ± **0**.78*
	Caudal neck	Proximal (3)	**27**.**98** ± **6**.**19**	**17**.**95** ± **2**.95*
		Distal (4)	**12**.**15** ± **1**.**44**	**9**.**71** ± **1**.41***

Nerve conduction velocity (NCV) and latency for proximal and distal portions of the left and right recurrent laryngeal nerves. Sites of supramaximal stimulation illustrated in Fig. 1 are shown in parentheses. Data are mean ± standard deviation. (***p < 0.001, *p < 0.05).

**Table 3:** Nerve conduction velocity and compound motor action potential amplitude of the recurrent laryngeal nerves.

			Horses without PSA		Horses with PSA	
			Left	Right	Left	Right
NCV (m/sec)		Proximal	69.05 ± 15.57	61.91 ± 8.63	64.49 ± 15.3	75.17 ± 4.21
		Distal	**43**.**82** ± **5**.**25**	**54**.**51** ± **10**.8**	**39**.**06** ± **6**.**61**	**52**.**43** ± **11**.45*
CMAP (uV)	Cranial neck	Proximal (1)	1329.92 ± 1059.09	1692.01 ± 1321.71	1467.8 ± 1871.85	2350.48 ± 956.55
		Distal (2)	1865.18 ± 1673.50	2422.99 ± 1343.69	**1527**.**17** ± **1125**.**68**	**2707**.**44** ± **1304**.93*
	Caudal neck	Proximal (3)	2118.1 ± 1336.91	1532.27 ± 1403.09	1348.91 ± 1170.89	2702.9 ± 1372.46
		Distal (4)	2924.89 ± 1622.8	2240.85 ± 1452.4	2624.64 ± 1823.45	2553.57 ± 1695.12

Nerve conduction velocity (NCV) and compound motor action potential (CMAP) amplitude data for proximal and distal portions of the left and right recurrent laryngeal nerves. Animals are categorized based on the presence of *pathologic spontaneous activity* (PSA) in the dorsal cricoarytenoid muscles. Sites of supramaximal stimulation illustrated in Fig. 1 are shown in parentheses. Data are mean ± standard deviation. (*p < 0.05, **p < 0.01, n = 10).

Lower NCV of the distal RLn was associated with the presence of *pathologic spontaneous activity* of the DCA muscle (p < 0.001, two-tailed Fisher’s Exact Test). No significant difference between horses with or without PSA was found regarding CMAP amplitude, while the left distal RLn showed lower NCV in horses with PSA (p = 0.0458, one-tail *t*-test, Table 3).

No significant effects of age or sex (p > 0.05, Pearson’s correlation coefficient and independent *t*-test). were observed in EMG or NCV data.

## Discussion

This study evaluated the electrophysiological characteristics of the sole laryngeal abductor muscle and its innervation in horses with normal laryngeal function.

We observed that the distal portions of the RLn conduct at a slower velocity bilaterally than do the proximal (vagal) portions. The difference in NCV between proximal and distal portions of a nerve has previously been reported in humans and dogs in several nerves, including the RLn^27,33,34^. This is attributed to the physiologic reduction in axon diameter, myelin thickness and internodal length that occur along the course of a nerve^35^. Similar to the findings from Steiss *et al*.^12^, and as reported in humans^28^, CMAP amplitudes were found to be lower after proximal (compared to distal) RLn stimulation, an effect attributed to the increasing conduction distance and temporal dispersion of MUPs^22^.

Distal reduction in conduction velocity has also been demonstrated in the equine radial and median nerves^36,37^ but the distal portions of the equine left and right RLn are not symmetrical in their conduction velocity. Previous work evaluating NCV of the RLn in mammals with shorter neck length (dog and human) have identified a small, but not significant, reduction in left-sided NCV^33,38^, while horses showed significantly reduced NCV in the distal portion of the left RLn for which the associated neuromuscular junctions are approximately 2 m from the cell body^39^.

Nerve conduction velocity is determined by myelinated fiber diameter, internodal length and myelin thickness^22,40^. The significantly lower NCV in the left distal RLn is suggestive of smaller fiber diameter, demyelination, reduced internode separation and/or myelin thickness in the left distal RLn compared to the right. Unfortunately, the major limitation of this study is the lack of morphometric evaluation of the RLn in this horse population. Previous studies investigated the RLn’s morphometry in horses, revealing fewer myelinated fibers and lower mean fiber diameter in the left distal RLn compared to the right^5,41^. Besides what seems to be a physiologic asymmetry in the RLn morphometry, that would explain the lower left distal NCV in our population, a significant progressive distal decrease in the total number of myelinated nerve fibers has been described in horses affected by Recurrent Laryngeal Neuropathy (RLN)^5,8^. RLN is a degenerative disorder of the RL nerves in horses, characterized by distal axonopathy, with segmental and paranodal demyelination and remyelination, that involves multiple cycles of denervation/reinnervation inducing fiber-type grouping and ultimately atrophy of the intrinsic laryngeal muscles^5–9,42^.

Despite the current reference standard to diagnosed RLN rely on endoscopic evaluation of laryngeal function at rest and during exercise^43–47^, the presence of neuropathologic changes in RLn and intrinsic laryngeal muscles in clinically normal horses has been extensively reported^5,8,42,48^. The existence of “sub-clinically” affected horses, whose prevalence is still unclear but reported as high as 30%^8^, should be considered in the interpretation of our findings; indeed, or data suggests the possibility of much higher subclinical disease prevalence.

A previous study on qualitative electromyographic evaluation of the DCA muscle in horses clinically affected by RLN, found PSA in the form of fibrillation potentials, positive sharp waves and *bizarre* high frequency discharge^16^. PSA is indicative of ongoing denervation^18,22^ and the equine DCA muscle showed fibrillation potentials after denervation^49^. Our study detected PSA in the left DCA muscle in 45% of the horses with normal laryngeal function, again emphasizing the likely high prevalence of subclinical disease.

The overall population also showed significantly higher values of MUPs Size Index and duration in the left DCA muscle. MUP duration reflects density, area and firing synchrony of muscle fibers within the motor unit and an increase in MUP duration is commonly found in chronic neurogenic disorders with reinnervation, that lead to an increased number of fibers per motor unit^18,22^. The finding in our dataset of left side significantly larger MUPs, based on longer duration and higher Size Index, is suggestive of larger motor units in the left compared to the right DCA muscle. Increased Size Index also discriminates neuropathic from normal muscle and is correlated to fatty degeneration of muscles affected by axonal neuropathy^23,50^. Horses sub-clinically affected by RLN show progressive neuropathic changes in the intrinsic laryngeal muscle correlated to the extent of damage in the distal left recurrent laryngeal nerve^5,51^. The muscles undergo cycles of denervation and reinnervation with initially compensatory enlargement of the remaining motor units through axonal sprouting that results in fiber type grouping and hypertrophy^51,52^. These early histopathologic changes provide a likely explanation of the larger MUPs recorded on the left side in our population, but without histologic assessment of the muscles this remains a hypothesis.

This study describes the electrophysiologic characteristics of the RL nerve and DCA muscle in clinically normal horses. We detected significant asymmetry in NCV and motor unit potential characteristics. Whilst these finding might represent physiologic variation, with the concurrent detection of electromyographic *pathologic spontaneous activity*, the data raises the question that this electrophysiologic asymmetry is a consequence of early stage RLN. The detection of electromyographic changes compatible with RLN in clinically unaffected horses is consistent with previous studies that identified “sub-clinical” subjects, presenting normal laryngeal function despite neuropathologic changes on nerve and muscle histological assessment.

## Data Availability

The datasets generated and analyzed during the current study are available from the corresponding author on reasonable request.
